# Capsazepine inhibits JAK/STAT3 signaling, tumor growth, and cell survival in prostate cancer

**DOI:** 10.18632/oncotarget.10775

**Published:** 2016-07-22

**Authors:** Jong Hyun Lee, Chulwon Kim, Seung Ho Baek, Jeong-Hyeon Ko, Seok Geun Lee, Woong Mo Yang, Jae-Young Um, Gautam Sethi, Kwang Seok Ahn

**Affiliations:** ^1^ College of Korean Medicine, Kyung Hee University, Seoul 130-701, Republic of Korea; ^2^ Department of Pharmacology, Yong Loo Lin School of Medicine, National University of Singapore, Singapore 117597

**Keywords:** capsazepine, STAT3, PTPε, apoptosis, prostate cancer

## Abstract

Persistent STAT3 activation is seen in many tumor cells and promotes malignant transformation. Here, we investigated whether capsazepine (Capz), a synthetic analogue of capsaicin, exerts anticancer effects by inhibiting STAT3 activation in prostate cancer cells. Capz inhibited both constitutive and induced STAT3 activation in human prostate carcinoma cells. Capz also inhibited activation of the upstream kinases JAK1/2 and c-Src. The phosphatase inhibitor pervanadate reversed Capz-induced STAT3 inhibition, indicating that the effect of Capz depends on a protein tyrosine phosphatase. Capz treatment increased PTPε protein and mRNA levels. Moreover, siRNA-mediated knockdown of PTPε reversed the Capz-induced induction of PTPε and inhibition of STAT3 activation, indicating that PTPε is crucial for Capz-dependent STAT3 dephosphorylation. Capz also decreased levels of the protein products of various oncogenes, which in turn inhibited proliferation and invasion and induced apoptosis. Finally, intraperitoneal Capz administration decreased tumor growth in a xenograft mouse prostate cancer model and reduced p-STAT3 and Ki-67 expression. These data suggest that Capz is a novel pharmacological inhibitor of STAT3 activation with several anticancer effects in prostate cancer cells.

## INTRODUCTION

Signal transducer and activator of transcription (STAT) proteins are involved in many biological responses and influence cell growth, survival, and metastasis [1, 2]. For example, STAT3, which is hyper-activated in a number of human malignancies, regulates the expression of numerous oncogenic genes that promote tumor progression [3, 4]. Constitutively activated STAT3 also promotes survival by increasing the transcription of anti-apoptotic genes (*Bcl-xL* and *Survivin*) and genes involved in cell cycle progression (*c-myc* and *Cyclin D1*), angiogenesis (VEGF and HIF-1α), and immune evasion (RANTES) [5, 6].

STAT3 activation is often deregulated in cancerous tissues, but not in normal margin tissues, in pathological specimens obtained via prostatectomy [7]. Abdulghan *et al*. detected phospho-STAT3 expression in 77% of lymph node and 67% of bone metastases in human prostate cancer patients, indicating that STAT3 is important for metastatic dissemination [8]. Mora *et al*. used antisense STAT3 oligonucleotides to reduce intracellular STAT3 protein levels in DU145 cells and found that cell growth decreased while apoptosis increased [9]. Azare *et al*. also found that constitutive STAT3 activation was associated with tumorigenesis and accelerated prostate epithelial cell migration [10]. Collectively, these data suggest that aberrant STAT3 activation occurs often in prostate cancer and is strong predictor of poor prognosis in patients.

Protein tyrosine phosphatase (PTP) ε (PTPε) exists in a transmembrane (PTPε M) form and a cytosolic (PTPε C) form, both of which are generated by alternative promoter usage in a single gene [11]. Tanuma *et al*. first reported that overexpression of PTPε C reduced IL-6-induced tyrosine phosphorylation of JAK1, Tyk2, gp130, and STAT3 [12]. Interestingly, PTPε C downregulates the IL-6- and IL-10-induced JAK/STAT signaling cascade in murine leukemic cells [13]. In humans, PTPε C mRNA expression was highest in peripheral blood leucocytes, and most normal tissues, including prostate, had lower expression [14]. PTPεC overexpression suppresses tumor incidence in the spleen, indicating that PTPεC may suppress tumorigenesis more generally [15].

In this study, we investigated the possible antitumor effects of capsazepine (Capz), a synthetic analogue of capsaicin with anti-proliferative effects in human PC-3 prostate cancer cells [16]. Capsaicin also has anticancer effects in different types of prostate cancer cells and mouse models [17–21]. In human MG63 osteosarcoma cells, Capz administration rapidly increased [Ca^2+^] and reduced tumor cell multiplication [22]. Additionally, Capz potentiates the antinociceptive effects of morphine in mice [23]. Luqman *et al*. demonstrated that Capz blocks TNFα-induced NF-κB activation and aromatase activity using computer-aided molecular docking studies [24]. More specifically, Capz increases TRAIL-induced apoptosis in colorectal cancer cells via ROS-JNK-CHOP-mediated upregulation of death receptors [25]. Here, we investigated the antitumor role of Capz in human prostate cancer cell lines and a xenograft mouse model and the molecular mechanisms underlying its effects.

## RESULTS

**Figure 1:**
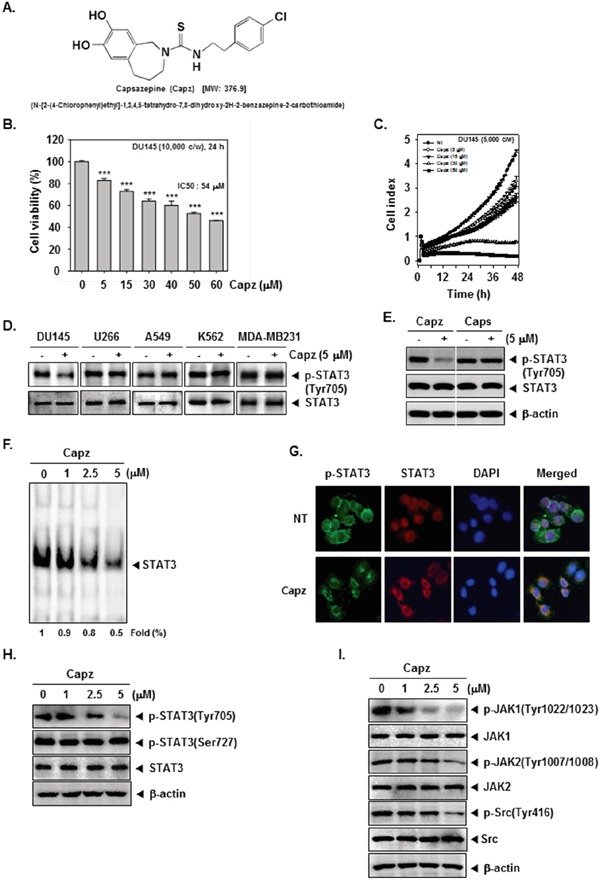
Capz inhibits the STAT3 signaling pathway by inhibiting constitutive JAK1/2 and Src activation **A**. The chemical structure of Capsazepine (Capz). **B**. DU145 cells (1 × 10^4^ cells/well) were treated with the indicated concentrations of Capz for 24 h and cell viability was determined by MTT assay. **C**. The cell proliferation assay was performed using the Roche xCELLigence Real-Time Cell Analyzer (RTCA) DP instrument (Roche Diagnostics GmbH, Germany). DU145 cells (5 × 10^3^ cells/well) were seeded onto 16-well E-plates and continuously monitored using impedance technology. **D**. DU145, U266, A549, K562, and MDA-MB231 cells (1 × 10^6^ cells/well) were treated with Capz (5 μM) for 6 h. Whole-cell extracts were prepared and immunoblotted with antibodies for p-STAT3(Tyr705) and STAT3. **E**. DU145 cells (1 × 10^6^ cells/well) were treated with Capz or Caps (5 μM) for 6 h. Whole-cell extracts were prepared and immunoblotted with antibodies for p-STAT3(Tyr705) and STAT3. **F**. DU145 cells (1 × 10^6^ cells/well) were treated with the indicated concentrations of Capz for 6 h and nuclear STAT3 levels were measured using EMSA. **G**. Capz inhibited phosphorylation and translocation of STAT3 to the nucleus. DU145 cells (4 × 10^4^ cells/well) were incubated with or without 5 μM Capz for 6 h and intracellular p-STAT3 and STAT3 distributions were analyzed by immunocytochemistry. **H**. DU145 cells (1 × 10^6^ cells/well) were treated with 0, 1, 2.5, or 5 μM Capz for 6 h. Whole-cell extracts were prepared and immunoblotted with antibodies for p-STAT3(Tyr705) and p-STAT3(Ser727). The same blots were stripped and reprobed with STAT3 antibody to verify equal protein loading. **I**. Equal amounts of lysates were analyzed by Western blot using antibodies against p-JAK1(Tyr1022/1023), p-JAK2(Tyr1007/1008), and p-Src(Tyr416). The same blots were stripped and reprobed with JAK1, JAK2, and Src antibodies to verify equal protein loading.

We investigated the effect of Capz on STAT3 activation and downstream proteins in human prostate cancer cells and a xenograft mouse model. The structure of Capz is shown in Figure 1A.

### Capz is cytotoxic to, and suppresses the proliferation of, prostate cancer cells

We first investigated the cytotoxic effects of Capz in DU145 cells. Capz reduced DU145 cell viability with an IC_50_ = 54 μM (Figure 1B). DU145 cells were then treated with the indicated concentrations of Capz, and cell viability was analyzed using an xCELLigence RTCA DP Instrument. As shown in Figure 1C, Capz suppressed DU145 cell proliferation in a dose- and time-dependent manner.

### Capz inhibits constitutive STAT3 phosphorylation in DU145 cells

We next examined whether Capz suppressed constitutive STAT3 activation in DU145, U266, A549, K562, and MDA-MB231 cells, all of which show constitutive STAT3 activation [26]. Interestingly, Capz suppressed STAT3 activation in DU145, but not in U266, A549, K562, or MDA-MB231, cells (Figure 1D, upper panel), and did not affect STAT3 expression in any of the cells (Figure 1D, lower panel). We then examined whether capsaicin (Caps) similarly inhibited constitutive STAT3 activation in human prostate cancer DU145 cells. Western blotting data showed that, while 5 μM Capz completely suppressed constitutive STAT3 activation, the same concentration of Caps did not affect STAT3 activation. Neither Capz nor Caps altered STAT3 expression (Figure 1E).

### Capz inhibits binding of STAT3 to DNA

We also examined whether Capz reduced STAT3 activity by measuring DNA binding. EMSA data demonstrated that Capz blocked STAT3-DNA binding in a concentration-dependent manner (Figure 1F), indicating that Capz substantially reduces the ability of STAT3 to bind to DNA.

### Capz inhibits both STAT3 phosphorylation and nuclear translocation

Next, we examined whether Capz inhibited the translocation of p-STAT3 and STAT3 into nuclei using immunocytochemistry. Capz substantially reduced both the phosphorylation and nuclear translocation of STAT3 (Figure 1G). Because STAT3 has two major phosphorylation sites, Tyr705 and Ser727, we evaluated whether Capz reduced phosphorylation at both sites. In DU145 cells, Capz inhibited Tyr705, but not Ser727, phosphorylation in a concentration-dependent manner. Finally, Capz did not affect overall STAT3 expression (Figure 1H).

### Capz inhibits constitutively active JAK1, JAK2, and Src kinases

To investigate the effect of Capz on constitutive JAK1 and JAK2 activation in DU145 cells, we measured phosphorylation of these kinases. As shown in Figure 1G, incubation with Capz inhibited JAK1 and JAK2 phosphorylation in a concentration-dependent manner. We also evaluated the effect of Capz on constitutive Src kinase activation in DU145 cells; Capz inhibited constitutive c-Src phosphorylation in a concentration-dependent manner (Figure 1I).

### Capz-induced inhibition of constitutive STAT3 activation is tyrosine phosphatase-dependent

**Figure 2:**
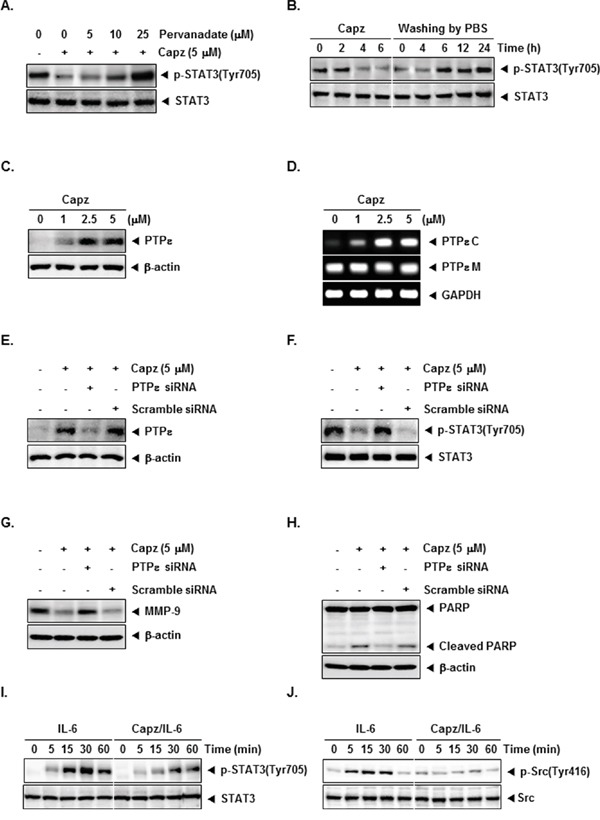
Capz increases protein tyrosine phosphatase epsilon (PTPε) levels in prostate cancer cells **A**. DU145 cells (1 × 10^6^ cells/well) were treated with the indicated concentrations of pervanadate and 5 μM Capz for 6 h. Whole-cell extracts were prepared and immunoblotted with antibody for p-STAT3(Tyr705). The same blots were stripped and reprobed with STAT3 antibody to verify equal protein loading. The results shown are representative of two independent experiments. **B**. DU145 cells (1 × 10^6^ cells/well) were treated with 5 μM Capz for the indicated durations (left) or treated for 6 h and washed with PBS twice to remove Capz before resuspension in fresh medium (right). Cells were removed at indicated times and lysed to prepare whole-cell extracts. **C**. DU145 cells (1 × 10^6^ cells/well) were treated with the indicated concentrations of Capz for 6 h. Whole-cell extracts were prepared and immunoblotted with antibody for PTPε. The same blots were stripped and reprobed with β-actin antibody to verify equal protein loading. The results shown are representative of two independent experiments. **D**. DU145 cells (1 × 10^6^ cells/well) were treated with the indicated concentrations of Capz for 6 h. Total RNA was extracted and PTPε C and PTPε M expression were examined by Reverse Transcription-Polymerase Chain Reaction (RT-PCR). Glyceraldehyde-3-phosphate dehydrogenase (GAPDH) was used as an internal control to verify equal RNA loading. **E**. DU145 cells (1 × 10^6^ cells/well) were transfected with either scrambled or PTPε-specific siRNA (50 nM). After 48 h, cells were treated with 5 μM Capz for 6 h. Whole-cell extracts were prepared and immunoblotted with antibody for PTPε. The same blots were stripped and reprobed with β-actin antibody to verify equal protein loading. The results shown are representative of three independent experiments. **F**. Equal amounts of lysates were analyzed by Western blot using antibody against p-STAT3(Tyr705). The same blots were stripped and reprobed with STAT3 antibody to verify equal protein loading. **G and H**. DU145 cells (1 × 10^6^ cells/well) were transfected with either scrambled or PTPε-specific siRNA (50 nM). After 48 h, cells were treated with 5 μM Capz for 24 h. Whole-cell extracts were prepared and immunoblotted with antibodies for MMP-9 and PARP. The same blots were stripped and reprobed with β-actin antibody to verify equal protein loading. **I**. LNCaP cells (1 × 10^6^ cells/well) were treated with 5 μM Capz for 6 h and then stimulated with IL-6 (25 ng/mL) for the indicated times. Whole-cell extracts were prepared and immunoblotted with antibodies for p-STAT3(Tyr705) and STAT3. **J**. Equal amounts of lysates were analyzed by Western blot using antibodies against p-Src(Tyr416) and Src.

Sodium pervanadate (a broad-acting tyrosine phosphatase inhibitor) largely reversed Capz-induced inhibition of constitutive STAT3 activation in DU145 cells (Figure 2A), indicating that this effect of Capz is dependent on at least one tyrosine phosphatase.

### Cells recovered from Capz-induced inhibition of STAT3 phosphorylation

We next investigated whether cells were able to recover from Capz-induced inhibition of STAT3 phosphorylation. DU145 cells were incubated with Capz for different lengths of time and then rinsed twice with PBS to remove Capz. The cells were then incubated with fresh medium for different intervals, after which STAT3 phosphorylation was measured. Capz again inhibited STAT3 phosphorylation (Figure 2B, left), but phosphorylated STAT3 expression gradually returned to baseline levels within 6 h after Capz was removed (Figure 2B, right). STAT3 protein levels did not change after Capz treatment or during recovery (Figure 2B, bottom).

### Capz increases PTPε protein and mRNA levels in DU145 cells

Protein tyrosine phosphatase (PTP) ε (PTPε), a member of the PTP family that exists in two forms (transmembrane: PTPε M, and cytosolic: PTPε C or cyt-PTPε) generated from a single gene by alternative promoter usage, is a negative regulator in STAT3 signaling pathway [27]. Capz clearly increased PTPε expression (Figure 2C), and substantially increased PTPε C, but not PTPε M, mRNA levels, in DU145 cells (Figure 2D).

### PTPε knockdown reverses Capz-induced inhibition of STAT3 activation and attenuates apoptosis

We then used siRNA to inhibit PTPε expression to confirm that Capz inhibited constitutive STAT3 activation via PTPε. The Capz-induced increase in PTPε expression was largely reversed in cells transfected with PTPε siRNA; PTPε expression did not change in cells transfected with scrambled siRNA (Figure 2E). Furthermore, Capz did not inhibit constitutive STAT3 activation in cells transfected with PTPε siRNA (Figure 2F). These results strongly support an important role for PTPε in Capz-induced inhibition of STAT3 phosphorylation. Moreover, as shown in Figure 2G and H, transfection with PTPε siRNA attenuated Capz-induced changes in MMP-9 levels and PARP cleavage compared to the control group, indicating that PTPε is a major putative target involved in both Capz-induced apoptosis and the regulation of MMP-9.

### Capz blocks IL-6-induced STAT3 and Src phosphorylation

Next, we examined whether Capz inhibited IL-6-induced STAT3 [28] and Src phosphorylation. LNCap cells, which lack constitutively active STAT3 and Src, were stimulated with IL-6 for various periods of time before STAT3 and Src phosphorylation were measured. IL-6 increased STAT3 and Src phosphorylation within 5-15 minutes, and Capz inhibited this increase(Figure 2I and 2J).

### Capz inhibits prostate cancer cell invasion

**Figure 3:**
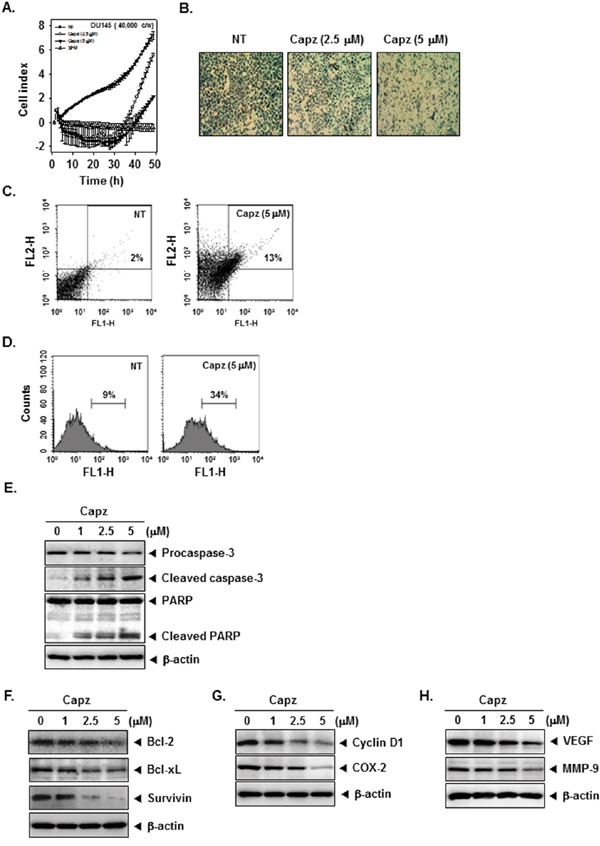
Capz inhibits invasion and promotes apoptosis in prostate cancer cells **A**. An invasion assay was performed using the Roche xCELLigence Real-Time Cell Analyzer (RTCA) DP instrument (Roche Diagnostics GmbH, Germany). DU145 cell invasion activity (4 × 10^4^ cells/well) was measured in a matrigel-coated CIM (cellular invasion/migration)-Plate 16 with the indicated concentrations of Capz. **B**. DU145 cells were seeded in a matrigel invasion chamber overnight in the absence of a serum, incubated with the indicated concentrations of Capz for 24 h, and then subjected to the invasion assay. Representative photographs of stained cells on the lower side of matrigel membrane from one of the three experiments are shown. **C**. DU145 cells (1 × 10^6^ cells/well) were treated with 5 μM of Capz for 24 h. The cells were incubated with a FITC-conjugated Annexin V antibody and then analyzed by flow cytometry. **D**. DU145 cells (1 × 10^6^ cells/well) were seeded into 6 well plates and treated with 5 μM of Capz for 24 h. The cells were fixed and incubated with TUNEL reaction solution and then analyzed by flow cytometry. **E**. DU145 cells (1 × 10^6^ cells/well) were treated with the indicated concentrations of Capz for 24 h. Whole-cell extracts were prepared; 20 μg portions of those extracts were resolved via 10% SDS-PAGE and probed with pro-caspase-3, cleaved caspase-3, and PARP antibodies. The same blots were stripped and reprobed with β-actin antibody to verify equal protein loading. **F**. Equal amounts of lysates were analyzed by Western blot using antibodies against Bcl-2, Bcl-xl, and Survivin. The same blots were stripped and reprobed with β-actin antibody to verify equal protein loading. **G**. Equal amounts of lysates were analyzed by Western blot using antibodies against Cyclin D1 and COX-2. The same blots were stripped and reprobed with β-actin antibody to verify equal protein loading. **H**. Equal amounts of lysates were analyzed by Western blot using antibodies against VEGF and MMP-9. The same blots were stripped and reprobed with β-actin antibody to verify equal protein loading. The results shown are representative of two independent experiments.

To examine whether Capz inhibited cell invasion *in vitro*, DU145 cells were seeded in the upper chamber of a matrigel invasion chamber in the absence of serum, and their ability to pass through extracellular matrix on a matrigel-coated membrane was measured. As shown in Figure 3A and 3B, Capz (2.5 and 5 μM) decreased invasion in DU145 cells compared to untreated cells.

### Capz promotes apoptotic cell death

We used an annexin V assay to evaluate the ability of Capz to induce apoptosis. The proportion of early apoptotic DU145 cells increased from 2% in untreated cells to 13% after treatment with 5 μM Capz (Figure 3C). TUNEL staining confirmed this increase in the induction of apoptosis; the proportion of TUNEL-positive cells increased from 9% in untreated cells to 34% in Capz-treated DU145 cells (Figure 3D). We also examined whether Capz altered procaspase-3 levels and PARP cleavage in DU145 cells. The procaspase-3 band was replaced by bands corresponding to cleaved forms after Capz treatment. Capz also increased PARP cleavage in a concentration-dependent manner. These results suggest that Capz promotes caspase-3-dependent apoptosis in DU145 cells (Figure 3E).

### Capz decreases levels of various oncogenic proteins

STAT3 activation controls the expression of various gene products involved in cell survival, proliferation, and angiogenesis. Capz treatment reduced levels of the anti-apoptotic proteins Bcl-2, Bcl-xL, and Survivin, the cell cycle regulator protein Cyclin D1, the inflammatory protein COX-2, the angiogenic protein VEGF, and the metastatic protein MMP-9 in a concentration-dependent manner (Figure 3F-3H).

### Antitumor effects of Capz in a xenograft prostate cancer model

**Figure 4:**
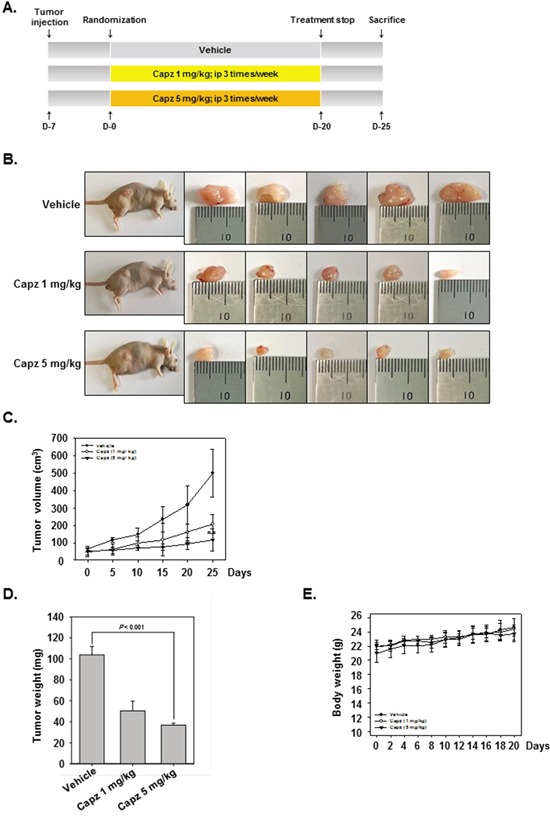
Antitumor effects of Capz in human prostate cancer xenograft mouse model **A**. Aschematic representation of the experimental protocol. DU145 cells (1× 10^7^ cells/mice) were injected subcutaneously into the right flanks of the mice. The animals were randomized into three groups 1 week after tumor cell injection based on tumor volume. Group I (control) was treated with PBS (100 μL i.p. 3 times/week), group II with Capz (1 mg/kg i.p. 3 times/week), and group III with Capz (5 mg/kg i.p. 3 times/week) (n = 8). **B**. Necropsy photographs of mice bearing subcutaneously implanted prostate tumors. **C**. Tumor diameters were measured every 5 days with Digimatic calipers, and tumor volumes were calculated using the formula V = 4/3 πr^3^ (n = 8). **D**. Tumor volumes (mean ± SE) based on tumor diameters measured on the last day of the experiment at autopsy using Digimatic calipers. **E**. Capz did not alter body weights, indicating the doses used were not toxic.

We then examined the ability of Capz to reduce the growth of human prostate cancer cells subcutaneously implanted in nude mice. One week after tumor cell injection, the animals were randomized into 3 treatment groups such that tumor volumes were similar among the groups. Treatment began 1 week after implantation and continued for up to 20 days (Figure 4A). Tumor diameters were measured every 5 days. Animals were killed 32 days after implantation and 25 days after the initiation of treatment, and the diameters of the excised tumors were measured. Tumor volumes increased more rapidly (Figure 4B and 4C), and tumor weights were higher (Figure 4D), in the vehicle control group than in the treatment groups. Capz treatment did not affect body weights (Figure 4E).

### Capz suppresses human prostate cancer growth *in vivo* and inhibits p-STAT3 and Ki-67 expression in tumor tissues

**Figure 5:**
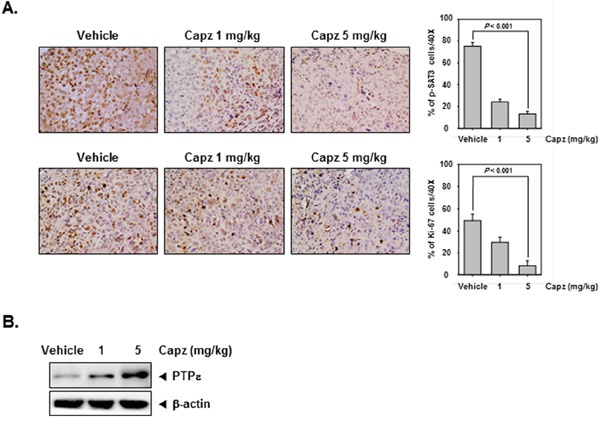
Capz reduces levels of oncogenic biomarkers in prostate tissues **A**. Immunohistochemical analysis indicated that Capz inhibited p-STAT3 expression compared to control group (Top panels). Percentages with positive staining for the given biomarkers are shown. The photographs were taken at 40× magnification. Immunohistochemical analysis of the proliferation marker Ki-67 indicated that Capz treatment inhibited prostate cancer cell proliferation in mice (bottom panels). **B**. Western blot analysis showed that Capz treatment reduced PTPε levels in whole cell extracts from mouse tissues. Western samples from three mice in each group were analyzed and representative data are shown.

We also administered Capz *in vivo* via intraperitoneal injection to evaluate its anti-cancer effects in mice subcutaneously injected with human DU145 prostate cancer cells. Immunohistochemical staining revealed that Capz decreased constitutive p-STAT3 expression in prostate tumor tissues compared to the control group (Figure 5A, upper panels). Capz also decreased Ki-67 expression in tumor tissues in a concentration-dependent manner (Figure 5A. lower panels).

### Capz induces PTPε expression in tumor tissues

We then measured PTPε protein levels in prostate tumors obtained from mice using Western blotting. As shown in Figure 5B, Capz increased PTPε protein levels in a concentration-dependent manner.

## DISCUSSION

The purpose of this study was to examine whether Capz inhibits STAT3 signaling cascades to inhibit the growth and survival of human prostate carcinoma cells. We found that Capz inhibited both constitutive and IL-6-induced STAT3 activation, and increased the expression of the receptor-like protein tyrosine phosphatase PTPε, in DU145 cells. Capz also reduced the levels of various oncogenic proteins, inhibited proliferation, induced apoptosis, and inhibited invasion in DU145 cells. Additionally, intraperitoneal injections of Capz inhibited tumor growth and STAT3 activation in tumor tissues from athymic *nu/nu* male mice with subcutaneous DU145 xenografts.

Here, we demonstrated for the first time that Capz inhibited both constitutive and IL-6-induced STAT3 phosphorylation specifically at tyrosine residue 705, and not at serine residue 727, in DU145 cells. Furthermore, these effects were cell-type specific; Capz did not inhibit STAT3 phosphorylation in U266, A549, K562, or MDA-MB231 tumor cells. Capz also reduced the binding of STAT3 to DNA and inhibited the activation of the protein tyrosine kinases JAK1, JAK2, and c-Src, which are upstream of STAT3, in DU145 cells. Recent reports indicate that increased constitutive and IL-6-induced STAT3 activation is common in prostate cancer cell lines and tissues [7, 9, 29, 30]. Furthermore, transfection of dominant-negative STAT3 plasmid or antisense STAT3 oligonucleotides inhibits STAT3 gene expression and promotes apoptosis in prostate cancer lines [7]. Additionally, Huang *et al*. demonstrated that stable constitutive STAT3 activation in benign prostatic epithelial cells induces transformation into aggressive prostate cancer cells [31].

We found that Capz-induced inhibition of STAT3 activation likely requires the activity of a PTP. Many PTPs, including SHP-1/2, TC-PTP, PTEN, PTP-1D, CD45, PTPε, and low molecular weight PTP, negatively regulate STAT3 signaling cascades [32]. Among these, cytosolic PTPε specifically results in negative feedback regulation of IL-6-induced JAK/STAT signaling when overexpressed in murine M1 cells [12, 13]. Indeed, we demonstrated for the first time that Capz increased PTPε protein and mRNA levels, likely contributing to the ability of Capz to inhibit constitutive STAT3 phosphorylation. Transfection with PTPε siRNA reversed the Capz-induced inhibition of STAT3 activation and increase in apoptosis, further suggesting that PTPε plays a critical role in Capz-induced inhibition of prostate cancer cell growth and survival. Capz also inhibits NF-κB activation in murine macrophages [33] and human embryonic kidney cells [24]. Whether the ability of Capz to inhibit STAT3 activation is also associated with the inhibition of NF-κB activation requires further investigation.

We also found that Capz dramatically reduced levels of the protein products of the following STAT3-regulated oncogenic genes: the anti-apoptotic proteins Bcl-2, Bcl-xL, and survivin, the cell cycle regulator protein cyclin D1, the pro-metastatic proteins MMP-9 and COX-2, and the angiogenic protein VEGF. Bcl-2 and Bcl-xL protein levels are commonly elevated in prostate cancer cells, protecting them from apoptosis [34] and reducing chemosensitivity [35]. The downregulation of Bcl-2, Bcl-xL, and survivin might be at least partially responsible for the ability of Capz to promote apoptosis in DU145 cells. Capz similarly downregulates these three anti-apoptotic proteins in human colorectal cancer HCT116 cells [25]. Finally, Capz treatment inhibited tumor growth in a xenograft prostate cancer model by inhibiting STAT3 phosphorylation and Ki-67 expression in tumor tissues. Collectively, our results indicate that Capz not only inhibits STAT3 signaling cascade activity by upregulating PTPε, but also inhibits prostate carcinoma tumor growth *in vivo*.

## MATERIALS AND METHODS

### Reagents

Capsazepine (Capz, Figure 1A) was purchased from Santa Cruz Biotechnology (Santa Cruz, CA). A Capz stock solution (100 mM) was prepared using dimethyl sulfoxide, stored at −80°C, and diluted in cell culture medium for use. RPMI 1640, fetal bovine serum (FBS), and an antibiotic-antimycotic mixture were obtained from Thermo Fisher Scientific Inc. (Waltham, MA). Trypan blue was obtained from GIBCO (Grand Island, NY). 3-(4,5-Dimethylthiazol-2-yl)-2,5-diphenyltetrazolium bromide (MTT) was purchased from Sigma-Aldrich (St. Louis, MO). SDS, Tris, Glycine, and NaCl were obtained from Sigma-Aldrich (St. Louis, MO). Bovine serum albumin was purchased from Biosesang (Sungnam, Korea). Rabbit polyclonal antibody against STAT3, mouse monoclonal antibodies against p-STAT3(Tyr705), p-STAT3(Ser727), p-STAT5(Tyr694), STAT5, Bcl-2, Bcl-xL, PTPε, Caspase-3, Cyclin D1, COX-2, VEGF, MMP-9, Survivin, PARP, and β-actin, goat anti-rabbit IgG-HRP, and goat anti-mouse IgG-HRP were purchased from Santa Cruz Biotechnology (Santa Cruz, CA). Antibodies against p-Src(Ty416), Src, p-JAK1(Tyr1022/1023), JAK1, p-JAK2(Tyr1007/1008), and JAK2 were purchased from Cell Signaling Technology (Beverly, MA). pMXS and pMXs-STAT3C were obtained from Addgene (Cambridge, MA). Whole-cell lysates of tumor tissues were prepared using T-PER Tissue Protein Extraction Reagent (Pierce, Rockford, USA).

### Cell lines and culture conditions

Human prostate carcinoma cells (DU145 and LNCap), human multiple myeloma U266 cells, human lung carcinoma A549 cells, human chronic myelogenous leukemia K562 cells, and human breast cancer MDA-MB231 cells were obtained from the American Type Culture Collection (Manassas, VA, USA). These cell lines were not authenticated by the authors. All cells were cultured in RPMI 1640 supplemented with 10% fetal bovine serum and 1% penicillin-streptomycin. Mouse embryonic fibroblasts (MEFs) were a kind gift from Professor Bharat B. Aggarwal at the M.D. Anderson Cancer Center, Houston, Texas, and were cultured in DMEM medium containing 10% fetal bovine serum and 1% penicillin-streptomycin. Cells were maintained at 37°C in a 5% CO_2_ atmosphere. Upon reaching ~70–90% confluence, cells were sub-cultured using 0.05% trypsin/EDTA (Gibco-BRL)

### Western blot analysis

Western blot analysis was performed as previously described [26].

### EMSA for STAT3-DNA binding

The electrophoretic mobility shift assay (EMSA) was performed as previously described [26].

### Immunocytochemistry for p-STAT3 and STAT3 localization

Immunocytochemistry was performed as previously described [26].

### Reverse transcription polymerase chain reaction (RT-PCR)

Reverse transcription polymerase chain reaction (RT-PCR) was performed as previously described [36].

### Transfection with PTPε siRNA and pMXs-STAT3C

The Neon™ Transfection System (Invitrogen, Carlsbad, CA) was used for transfection via electroporation. Transfection efficiency was measured using Western blot analysis. MEF cells were resuspended in 120 μL of Neon Resuspension Buffer R for every one million cells in preparation for transfection. For each electroporation, DU145 cells and 10 μL of PTPε siRNA (50 nM, Santa Cruz Biotechnology, Santa Cruz, CA) were aliquoted into a sterile microcentrifuge tube. MEF cells and 1 μg of pMXs and pMXs-STAT3C plasmids were aliquoted into a sterile microcentrifuge tube. A Neon Tip was inserted into the Neon Pipette and the cell-plasmids mixture was aspirated into the tip while avoiding the formation of air bubbles. The Neon Pipette was then inserted into the Neon Tube containing 3 mL of Neon Electrolytic Buffer E in the Neon Pipette Station. DU145 cells were subjected to a single 1,260-volt pulse with a width of 20. MEF cells were subjected to a single 1,350-volt pulse with a width of 30. 48 h after transfection, cells were treated with 5 μM Capz for 6 or 24 h, and whole-cell extracts were prepared for measurement of PTPε, p-STAT3(Tyr705), PARP, and β-actin by Western blot.

### Monitoring of cell growth with an RTCA DP Instrument

Monitoring of cell growth with an RTCA DP Instrument was performed using a previously described method [37].

### Invasion assay

The invasion assay was performed as previously described [38].

### Invasion assay with RTCA

The invasion assay with RTCA was performed using a previously described method [37].

### Cell cycle analysis

Cell cycle analysis was performed as previously described [36].

### Annexin V assay

The annexin V assay was performed using a previously described method [37].

### TUNEL assay

The TUNEL assay was performed as previously described [36].

### Mice and housing

All procedures involving animals were reviewed and approved by the Kyung Hee University Institutional Animal Care and Use committee [KHUASP(SE)-15-031]. Six-week-old athymic balb/c *nu/nu* male mice were purchased from Orientbio Inc. (Sungnam, Korea). The animals were housed (8 mice/cage) in standard plexiglass mouse cages in a room maintained at constant temperature and humidity under a 12 h light and dark cycle and fed regular autoclaved mouse chow with water *ad libitum*. Mice were allowed to acclimate to housing for 1 week before testing to ensure adaptation to the new environment. None of the mice exhibited any lesions and all tested pathogen-free.

### Subcutaneous implantation of DU145 cells

Subcutaneous implantation of DU145 cells was performed as previously described [26].

### Experimental protocol

One week after implantation, tumor diameters were measured using Digimatic calipers (Mitutoyo Company, Japan). When tumors reached 0.25 cm in diameter, the mice were randomized into the following treatment groups (n = 8/group) such that initial tumor volumes were similar among the groups. Group I (control) was treated with PBS (100 μL i.p. 3 times/week), group II with Capz (1 mg/kg i.p. 3 times/week), and group III with Capz (5 mg/kg i.p. 3 times/week) (n = 8). Treatment continued for up to 20 days from the date of randomization (Day 0). Tumor volumes were measured every 5 days. The mice were killed 25 days after randomization. Tumors were carefully excised and measured to calculate tumor volume using the formula V = 4/3 πr^3^, where r is the mean of the 3 dimensions (length, width, and depth). Half of the tumor tissue was fixed in formalin and embedded in paraffin for immunohistochemistry and routine hematoxylin and eosin (H&E) staining. The other half was snap-frozen in liquid nitrogen and stored at −80°C.

### Western blot analysis of tumor tissues

Western blotting of tumor tissues was performed as previously described [39].

### Immunohistochemical analysis of prostate tumor samples

Immunohistochemical analysis of prostate tumor samples was performed as previously described [39].

### Statistical analysis

All numeric values are shown as mean ± SD. Statistically significant differences between experimental and untreated control groups were detected using Student's unpaired *t*-tests; *p* < 0.05 was considered statistically significant.
